# Impaired Blood Dendritic Cell Numbers and Functions after Aneurysmal Subarachnoid Hemorrhage

**DOI:** 10.1371/journal.pone.0071639

**Published:** 2013-08-09

**Authors:** Antoine Roquilly, Cécile Braudeau, Raphael Cinotti, Erwan Dumonte, Rémi Motreul, Régis Josien, Karim Asehnoune

**Affiliations:** 1 Laboratoire UPRES EA 3826 "Thérapeutiques cliniques et expérimentales des infections", Faculté de Médecine, Université de Nantes, Nantes, France; 2 Service d’Anesthésie Réanimation chirurgicale - Hôtel Dieu, Centre Hospitalier Universitaire, Nantes, France; 3 INSERM Unité Mixte de Recherche 1064 “Centre de Recherche en Transplantation et Immunologie”, Nantes, France; 4 Institut de Transplantation –Urologie – Néphrologie, Centre Hospitalier Universitaire, Nantes, France; 5 Laboratoire d’Immunologie, Centre d’Immunomonitorage Nantes Atlantique, Centre Hospitalier Universitaire, Nantes, France; 6 Service d’Anesthésie Réanimation chirurgicale, Hôpital Guillaume et René Laennec, Centre Hospitalier Universitaire, Nantes, France; Hospital Nacional de Parapléjicos - SESCAM, Spain

## Abstract

**Previous Presentation:**

Portions of this study were presented at the Annual Congress of Société Française d’Anesthésie et de Réanimation in Paris, September 2012.

**Background:**

Toll-like receptor (TLR) agonists are promising therapy for the prevention of nosocomial infections in critical ill patients. We aimed to analyze the TLR-reactivity of circulating dendritic cells (DC) as assessed by cytokine production after an *ex vivo* challenge with TLR agonists in aneurysmal subarachnoid hemorrhage (SAH) patients.

**Methods and Findings:**

A single-center prospective observational study took place in one intensive care unit of a teaching hospital. Blood samples were harvested on days 2, 5 and 10 in 21 severe SAH patients requiring mechanical ventilation and 17 healthy controls. DC production of cytokines (Tumour Necrosis Factor, TNF-α; Interleukin, IL-12; and Interferon, IFN-α) was assessed by intracellular immunostaining on TLR-3, 4, 7/8 and 9 stimulations. SAH patients had decreased numbers of blood myeloid (mDCs) and plasmacytoid DCs (pDCs) on days 2, 5 and 10. Compared with the healthy controls, the frequency of mDCs producing TNF-α after TLR-3 stimulation was decreased in the SAH patients. The frequency of myeloid DCs producing IL-12 after TLR-3 and 4 stimulations was also decreased in the SAH patients. In contrast, the mDCs response to TLR-7/8 was not impaired in the SAH patients. The frequency of pDCs producing TNF-α^+^ and IFN-α^+^ on TLR-7/8 stimulation were reduced at all of the tested times in the SAH patients, whereas reactivity to TLR-9 was preserved. On day 2, the pDCs from non-survivor patients (n = 8) had a decreased ability to produce IFN-α on TLR-9 stimulation compared with the survivors.

**Conclusions:**

These data suggest functional abnormalities of circulating pDCs and mDCs that could be important for immunomodulation after SAH.

## Introduction

Acute aneurysmal subarachnoid hemorrhage (SAH) is a severe neurological condition [1]. More than 25% of the patients die during their hospital stay or present with long-term severe disabilities [2], [3]. In brain-injured patients, evidence suggests that brain injury negatively affects the cells of the innate and adaptive immune system, and that nosocomial infections and symptomatic complications develop as a consequence of a transient immunodepression [4]. A better comprehension of SAH-induced immunosuppression could therefore be useful to improve outcomes and the monitoring of patients.

The initial response to brain injuries is pro-inflammatory with a systemic inflammatory response syndrome (SIRS). However, patients with “septic like syndrome” like severe trauma or brain injury (stroke, traumatic brain injuries, SAH) also display signs of systemic immunosuppression (See [5], [6] for review). In brain-injured patients, blood T-lymphocytes show decreased cytokine production capacities in vitro [7], [8]. Also, NK cells count and functions are decreased [9], [10]. Interestingly, humoral immune response is less affected [11]. Finally, myeloid cells functions are also impaired, including decreased phagocytotic activity of granulocytes, and monocyte deactivation.

Dendritic cells (DCs) are specialise in pathogen recognition and are endowed with the unique capacity to activate naive T cells and to regulate T-cell differentiation [12]. DCs express many toll-like receptors (TLR) which are critical sensors of microbes that induce cytokine production by DCs, a mandatory step for full activation of adaptive immune response [13]. Previous studies have shown that DC numbers and maturation state were affected in several critical conditions [14]–[16], yet other critical DC functions such as TLR-reactivity and cytokine production were poorly described.

Obtaining knowledge regarding TLR-induced cytokine production by DC in SAH patients could provide opportunities for treating immunosuppression. Indeed, the stimulation of DC by TLR agonists is a promising therapy for the prevention of infectious diseases in humans without critical illness [17]. In a mice model reproducing immunosuppression, we have demonstrated that a TLR agonist can restore DC cytokine production and enhance lung response to pneumonia [18], [19]. However, a decreased capacity of patients’ leukocytes to produce cytokines *in vitro* after a TLR-4 challenge is one of the main feature of this immunosuppression in humans [20]. We thus hypothesised that cytokine production by DC upon TLR stimulation could be altered after SAH. This could be an important issue limiting the potential efficiency of immunomodulation using TLR-agonists in SAH patients. Our aim was therefore to describe the time evolution of circulating DC numbers and of the cytokine production of DCs on TLR stimulation after SAH.

## Materials and Methods

### Ethical Standards

An institutional review board for human experimentation approved the protocol (Comité de Protection des Personnes de Nantes, authorization number AC-2008-433/French Ministry of Health). Written informed consent from a next-of-kin was required for enrolment. Retrospective consent was obtained from patients when available. All experiments were in accordance with the declaration of Helsinki.

### Study Population

Patients were enrolled from June 2011 to June 2012 in a French surgical ICU of a university hospital. Inclusion criteria were: severe aneurysmal SAH with a WFNS score ≥4, and mechanical ventilation for more than 48 hours. Exclusion criteria were previous immunosuppression, cancer in the previous five years, treatment with corticosteroids, and pregnancy. Matched control samples were collected from 17 healthy donors after obtaining informed consent (sex, race).

### General Care for SAH Patients [1], [21]

Diagnosis of aneurysmal SAH was confirmed by a brain CT-scan. The aneurysm was secured during an arteriography with an endovascular coil in the first 24 hours. Ventriculostomy was performed by a neurosurgeon in case of hydrocephalus on CT. Patients were sedated with fentanyl and midazolam and were kept in a semirecumbent position. Sedation was stopped when patients were considered as low risk of intra-cranial hypertension. Intracranial pressure was monitored with an intraparenchymal probe (Codman, Johnson and Johnson Company, Raynham, Mass., USA.) in patients considered at increased risk of intracranial hypertension. Cerebral perfusion pressure (CPP) was maintained above 60 mmHg with boli of isotonic solutions and continuous infusion of norepinephrine. Nimodipine was administered for 21 days. Screening for vasospasm was performed once a day by trans-cranial Doppler (TCD) of the middle cerebral artery. An arteriography was performed whenever mean artery flow velocity assessed by TCD was 50% higher than on the first day, or above 120 cm.s^−1^ or in the case of an unexplained fever or a new neurologic deficit. Secondary brain injuries were prevented by avoiding hypoxaemia and anaemia (hemoglobin <10 g.dl^−1^), maintaining body temperature between 36.0°C and 37.0°C, ensuring normoglycaemia and normocapnia (between 4.6 and 5.5 kPa). Osmotherapy (Mannitol, bolus of 0.5 g.kg^−1^, repeatable once in case of poor ICP control) was used to control episodes of intracranial hypertension. When control of ICH was poor, sodium thiopental was used with a loading dose (2–3 mg.kg^−1^) followed by continuous administration (2–3 mg.kg^−1^.hr^−1^).

### Blood Sample Collection

Venous blood samples were collected in EDTA and heparin vacutainers and processed for analysis within 4 hours on days 2, 5 and 10 after SAH. Patient sera were frozen at −80°C.

### Detection of Cytokines in Serum of SAH Patients

Using a multiplex assay, levels of 8 cytokines and growth factors (IL-2, IL-4, IL-6, IL-8, IL-10, GM-CSF, IFN-γ, TNF-α, Bio-Rad, Marnes-la-Coquette, France) were investigated in sera collected from patients and healthy controls.

### Antibodies and Reagents

DCs were identified using 6-color flow cytometry assay as described previously [22], [23]. Briefly, whole blood samples were stained with the following antibodies: CD45-Amcyan, Lineage cocktail1-FITC, HLA-DR-APC-Cy7, CD11c-PECy7 and CD123-PECy5, all from BD Biosciences. TNF-APC (BD Biosciences), IL-12-efluor450 (eBiosciences, Paris, France) and IFNα-PE (Miltenyi Biotec) Abs were used to identify intracellular cytokines after stimulation with TLR ligands.

### Flow Cytometry was Performed on a BD FacsCanto II Analyzer with DIVA Software (BD Biosciences)

TLR ligands: Poly(I:C) (TLR-3-L, 100 µg/ml), CL097 (imidazoquinoline compound, TLR 7/8-L, 2 µg/ml) and CPG ODN2395 (Type C CPG oligonucleotide, TLR 9-L, 50 µM) were obtained from Invivogen (Toulouse, France) LPS (lipopolysaccharides from Escherichia coli O26:B6, TLR 4-L, 0.1 µg/ml) was purchased from Sigma-Aldrich (St Louis, MI). GolgiPlug and Cytofix/Cytoperm Plus were obtained from BD Biosciences.

### Cytokine Production by Dendritic Cells

Heparinated whole blood samples were incubated for 3h30 at 37°C under 5% CO2 conditions with TLR ligands for DC stimulation. GolgiPlug were added during the last 2h30 hours of incubation to inhibit cellular cytokine release. Control conditions included stimulation with medium alone as negative control. Whole blood samples were then incubated with surface mAbs for 15 min, followed by erythrocyte lysis (BD Biosciences). Samples were then fixed, permeabilised with Cytofix/Cytoperm Plus and stained with cytokine-directed mAbs.

### Statistics

Data are presented as plots with medians and interquartile range (IQR). Comparisons between groups (healthy donors versus SAH patients, or sub-group analysis) were made using the non-parametric Mann-Whitney test for continuous variables and the Fisher exact test for categorical variables. The Friedman test was used to assess variations between time points in SAH patients with the use of Dunn’s multiple comparison test for *post hoc* test. A *P*-value of less than 0.05 was considered statistically significant.

## Results

### Population

SAH patients and healthy controls are described in Table 1. Blood samples from 21 patients and 17 healthy controls were analyzed. The median world federation of neurological surgeons (WFNS) score and glasgow coma scale were 5 (4–5) and 6 (3–8) respectively. Twenty (95%) SAH patients had criteria for systemic inflammatory response on day 2. The median CRP level on day 2 was 58 (44–131) mg/dl. The median duration of mechanical ventilation was 18 (IQR, 11–24) days. Of 21 patients, 7 (33%) developed nosocomial pneumonia and 8 (38%) died in the intensive care unit (ICU).

**Table 1:** Characteristics of the study population.

	Healthy donors(n = 17)	Whole population(n = 21)	Sub-group analysis		
			Survivors(n = 13)	Non-survivors(n = 8)	*P*
Age (years), *median (IQR)*	42 (30–60)	56 (44–64)	58 (45–63)	54 (47–65)	0.72
Male, *N (%)*	8 (47)	9 (43)	6 (46)	3 (38)	0.68
Medical history, *N (%)*					
Smoking	/	7 (33)	7 (54)	0 (0)	0.02
Obesity	/	2 (10)	2 (15)	0 (0)	0.50
Chronic lung disease	/	0 (0)	0 (0)	0 (0)	1.00
Diabetes mellitus	/	0 (0)	0 (0)	0 (0)	1.00
Chronic kidney failure	/	0 (0)	0 (0)	0 (0)	1.00
Severity Scores on admission, *median (IQR)*					
SAPS II	/	47 (43–52)	49 (44–52)	47 (43–53)	0.86
Glasgow Coma Scale	/	6 (3–8)	5 (3–7)	6 (5–8)	1.00
WFNS score	/	5 (4–5)	4 (4–5)	5 (4–5)	0.23
SAH management and complications					
Osmotherapy, *N (%)*	/	11 (52)	7 (54)	4 (50)	0.86
Extraventricular drainage, *N (%)*	/	19 (90)	13 (100)	6 (75)	0.06
Barbiturates, *N (%)*	/	10 (48)	6 (46)	4 (50)	0.86
Vasospasm, *N (%)*	/	4 (19)	3 (23)	1 (13)	0.55
Cerebral angioplasty, *N (%)*	/	18 (86)	12 (92)	6 (75)	0.27
Craniotomy, *N (%)*	/	7 (33)	5 (38)	2 (25)	0.53
Norepinephrine (days), *median (IQR)*	/	6 (4–9)	7 (4–10)	3 (5–7)	0.32
Nosocomial Pneumonia - *N (%)*	/	7 (33)	5 (38)	2 (25)	0.53
ARDS - *N (%)*	/	3 (14)	2 (15)	1 (13)	0.85
Bacteriaemia - *N (%)*	/	2 (10)	2 (15)	0 (0)	0.24
Septic shock - *N (%)*	/	2 (10)	2 (15)	0 (0)	0.24
Mechanical ventilation support (days) – *mean (SD)*	/	20 (11)	20 (9)	17 (14)	0.20
Length of ICU stay (days) – *median (IQR)*	/	23 (12)	25 (9)	17 (14)	0.04
Death in ICU – *N (%)*	/	8 (38)	0 (0)	8 (100)	/

ARDS: acute respiratory distress syndrome, ICU: intensive care unit, SAH: subarachnoid hemorrhage, SAPS: simplified acute physiological score, WFNS: World Federation of Neurological Surgeons.

### Time-evolution of Blood Cytokine Levels in SAH Patients

**Figure 1:**
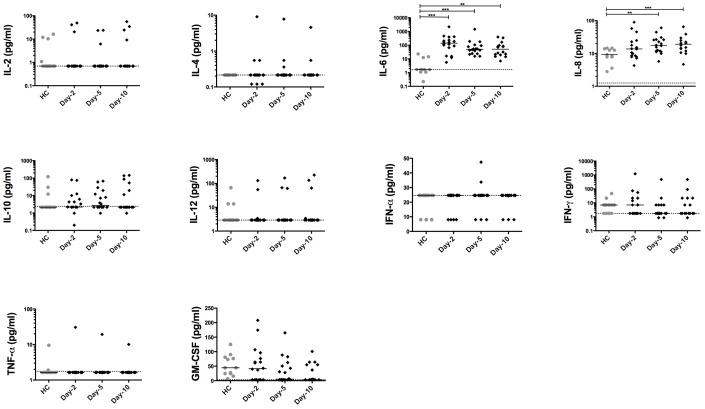
Time course of circulating cytokines in patients with aneurysmal subarachnoid hemorrhage. Blood samples were drawn from SAH patients (N = 18) and from HC (N = 11). The level of IL-2, -4, -6, -8, -10, -12, IFN-α, IFN-γ, TNF-α and GM-CSF were assessed in plasma by luminex on days 2, 5 and 10. Plots represent median (interquartile range). HC: healthy controls. SAH: aneurysmal subarachnoid hemorrhage. Dashed line indicates the lower limit of detection. * *P*<0.05, ** *P<*0.01.

We first investigated the blood levels of several cytokines. Surprisingly, except for IL-6 and IL-8 which were increased after SAH, the blood levels of the pro- and anti-inflammatory cytokine tested remained unchanged in SAH patients compared with healthy controls (Fig. 1). We found a trend toward a lower GM-CSF level on day-5 and day-10, although this did not reach statistical significance when compared with healthy controls.

### Decreased Numbers of Circulating Myeloid DCs and Plasmacytoid DCs in SAH Patients

**Figure 2:**
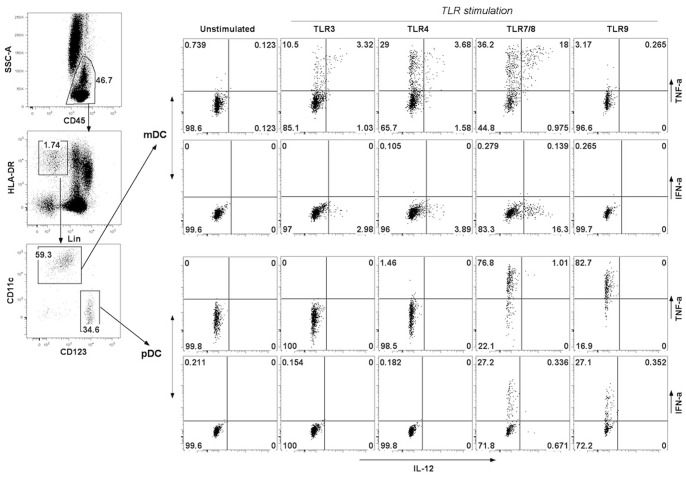
Gating strategy used to identify blood DC subsets and intracellular cytokines production in DCs in whole blood stimulated with TLR ligands. Whole blood samples were incubated with TLR3, 4, 7/8 or 9 ligands for 3.5-hour and then stained for identification of myeloid DC (HLA-DR+, Lin-, CD11c+, CD123-) and plasmacytoid DC (HLA-DR+, lin−, CD11c−, CD123+) together with intracellular cytokine production (TNFα, IL-12, IFNα).

**Figure 3:**
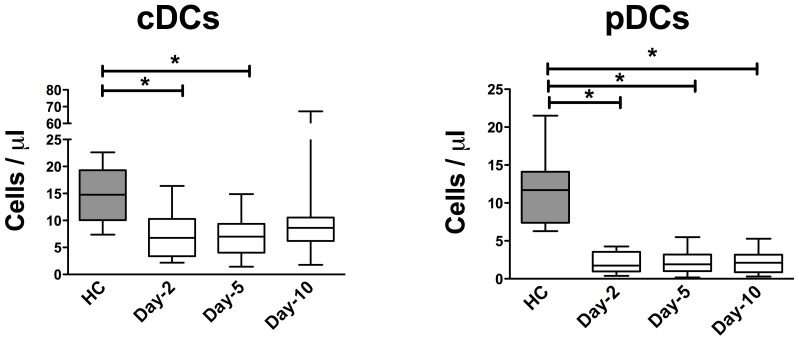
Time course of circulating mDCs and pDCs numbers in patients with aneurysmal subarachnoid hemorrhage. Comparison of circulating (A) myeloid DC and (B) plasmacytoid DC counts in SAH patients (N = 21) on days 2, 5 and 10 compared with HC (N = 11). Plots represent median (Interquartile ranges). HC: healthy controls. SAH: aneurysmal subarachnoid hemorrhage. DCs: dendritic cells. * *P*<0.05, ** *P*<0.01.

Peripheral blood DC subsets were quantified in whole blood by flow cytometry. The two main subsets of DC, myeloid DCs (mDCs) and plasmacytoids DCs (pDCs), can be separated on the basis of CD11c and CD123 expression: mDCs that are CD11c+ CD123- and pDCs that are CD11c-CD123+ (gating strategy, Fig. 2). The number of circulating mDCs was decreased on days 2 and 5 when compared with healthy controls (*P*<0.05 for both comparisons, Fig. 3a). For pDCs, the decrease was dramatic on days 2, 5 and 10 versus healthy controls (*P*<0.01 for all comparisons, Fig. 3b).

### Myeloid DCs Reactivity to TLR-3 and TLR 4 Agonists were Altered in SAH Patients

**Figure 4:**
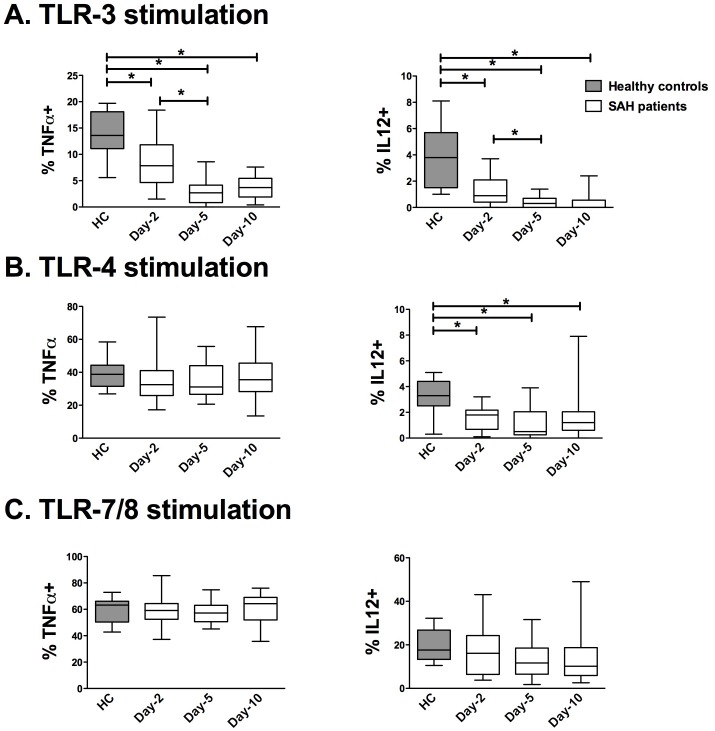
Time course of TLR-induced production of TNF-α and IL-12 in circulating mDCs from patients with aneurysmal subarachnoid hemorrhage. Intracellular cytokine measurement was performed in circulating mDCs from SAH patients (N = 21) on days 2, 5 and 10 and from HC (N = 11). The percentages of mDCs expressing TNF-α or IL-12 were assessed after a 3.5-hour *ex vivo* stimulation with (**A**) polyIC (TLR-3 agonist), (**B**) lipopolysaccharide (TLR-4 agonist) or (**C**) CL097 (TLR-7/8 agonist). The percentage of positive DCs without TLR-stimulation was below 1% (data not shown). The results are presented as percentages of mDCs expressing TNF-α (%TNF-α^+^) or IL-12 (% IL-12^+^). Plots represent median (Interquartile ranges). HC: healthy controls. mDCs: myeloid dendritic cells. SAH: aneurysmal subarachnoid hemorrhage. TNF-α: tumour necrosis factor -α. IL-12: intreleukin-12. * *P*<0.05.

Myeloid DCs express high levels of TLR 1, 2, 3, 4, 5, 6 and 8 [23], [24]. On TLR-stimulation, mDCs produce several cytokines that are key regulators of immune response to pathogens, notably: TNF-α induces the maturation of innate immune cells (such as monocytes and pDCs), and IL-12 activates natural killer cells and drives Th1 cell differentiation [24]. We therefore evaluated the ability of mDCs to produce TNF-α and IL-12 by flow cytometry (gating strategy, Fig. 2). After TLR-3 stimulation (polyI:C), the percentages of TNF-α^+^ mDCs and IL12^+^ mDCs were decreased in SAH patients compared with healthy controls (HC) on day 2 without recovery on days 5 and 10 (Fig. 4a). After TLR-4 stimulation (LPS), mDC ability to produce TNF-α was not altered in SAH patients compared with healthy controls, whereas the percentage of IL-12**^+^** mDCs was decreased at all of tested times (Fig. 4b). The percentages of TNF-α**^+^** and IL12**^+^** mDCs after stimulation by TLR-7/8 agonists were not altered in SAH patients as compared with the healthy controls (Fig. 4c).

### Plasmacytoid DCs Reactivity to TLR-7/8 Agonist was Altered in SAH Patients

**Figure 5:**
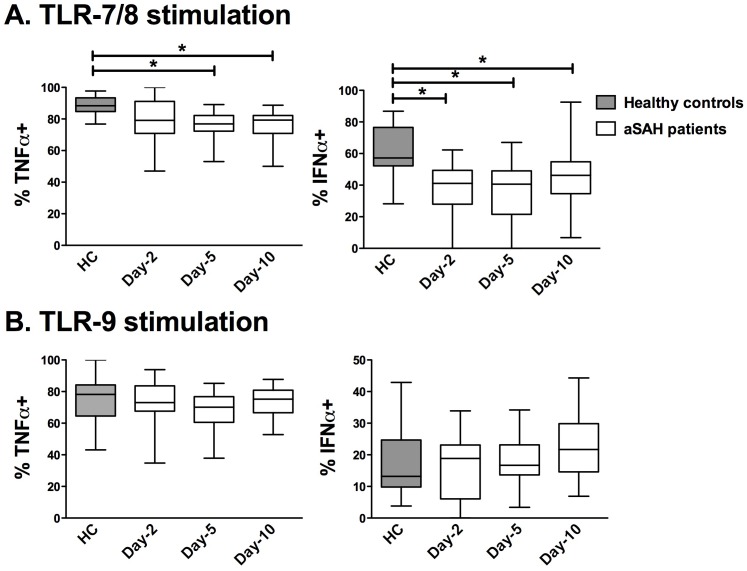
Time course of TLR-induced productions of TNF-α and IFN-α in circulating pDCs from patients with aneurysmal subarachnoid hemorrhage. Intracellular cytokine measurement was performed in circulating pDCs from SAH patients (N = 21) on days 2, 5 and 10 after brain injury and from HC (N = 11). The percentages of pDCs expressing TNF-α or IFN-α were assessed after a 3.5-hour *ex vivo* stimulation with (**A**) CL097 (TLR-7/8 agonist) or (**B**) CpG (TLR-9 agonist). The percentage of positive pDCs without TLR-stimulation was below 1% (data not shown). The results are presented as percentages of pDCs expressing TNF-α (%TNF-α^+^) or IFN-α (%IFN-α^+^). Plots represent median (Interquartile ranges). HC: healthy controls. IFN-α: interferon. pDCs: plasmacytoid dendritic cells. SAH: aneurysmal subarachnoid hemorrhage. TNF-α: tumour necrosis factor -α. *P<0.05.

Type I IFN has emerged as a central coordinator of innate and adaptive immune response since this cytokine induces the maturation of antigen-presenting cells [25]. Among DCs, activated pDCs are specialised in rapid and massive production of IFN-α [26], whereas resting pDCs may exhibit immunosuppressive properties [27]. We therefore investigated the production of TNF-α and IFN-α by pDCs after TLR-7/8 or TLR-9 stimulations (gating strategy Fig. 2). On TLR-7/8 stimulation, the percentages of TNF-α**^+^** pDCs were reduced on days 5 and 10 in SAH patients compared with healthy controls, whereas the percentage**s** of IFN-α**^+^** pDCs were decreased at all of the tested times (Fig. 5a). After TLR-9 stimulation, neither the percentage of TNF-α**^+^** nor the percentage of IFN-α**^+^** pDCs was altered in SAH patients compared with healthy controls (Fig. 5b).

### Sub-group Analysis of Survivors versus Non-survivors

**Figure 6:**
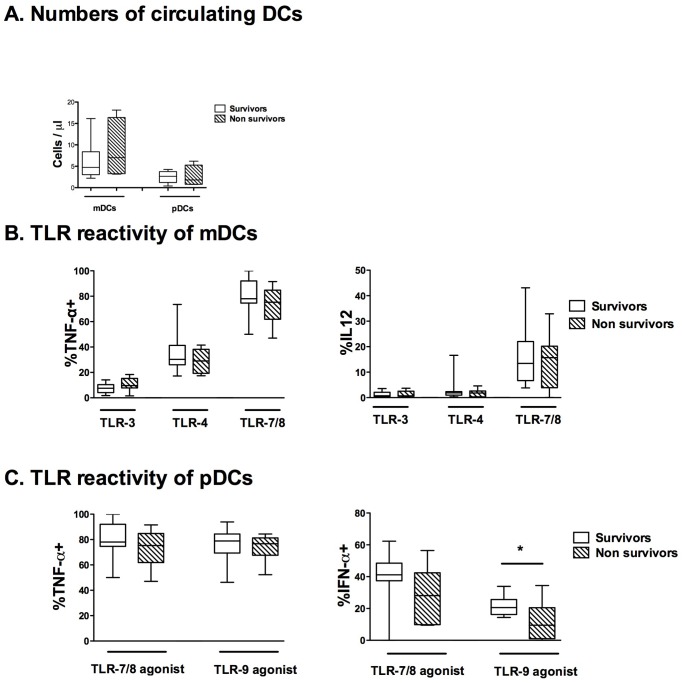
Exploratory comparison of mDC and pDC status on day 2 in survivors and non-survivors. (**A**) The number of circulating myeloid DCs and plasmacytoid DCs were compared on day 2 between 13 survivors and 8 non-survivors. On day 2, mDCs and pDCs were stimulated *ex vivo* with polyIC (TLR-3 agonist), lipopolysaccharide (TLR-4 agonist), CL097 (TLR-7/8 agonist) and CpG (TLR-9 agonist) for 3.5 hours. (B) The percentages of mDCs expressing TNF-α or IL-12 and (C) the percentages of pDCs expressing TNF-α or IFN-α were compared between survivors and non-survivors. The percentage of positive DCs without TLR stimulation was below 1% (data not shown). The results are presented as percentages of DCs expressing TNF-α (%TNF-α^+^), IL-12 (%IL-12^+^) or IFN-α (%IFN-α^+^). Plots represent median (Interquartile ranges). * *P*<0.05.

We performed a sub-group analysis comparing survivors and non-survivor SAH patients on day 2. No difference in demographical characteristics was apparent on ICU admission between survivors and non-survivors (Table 1). The circulating levels of cytokines were not different between survivors and non-survivors at any time (data not shown). The numbers of circulating mDCs and pDCs on day 2 were not altered in survivors compared with non-survivors (Fig. 6a). TNF-α^+^ and IL-12^+^ in mDCs after TLR stimulations were not different between survivors and non-survivors (Fig. 6b). The percentages of TNF-α^+^ pDCs were not different between survivors and non-survivors after TLR-7/8 or TLR-9 stimulation (Fig. 6c). The percentages of IFN-α^+^ pDCs on day 2 tended to be lower in SAH patients after TLR-7/8 (*P*  = 0.126) and were more significantly decreased in non-survivors than in survivors after TLR-9 stimulation (Fig. 6c).

## Discussion

Dendritic cells play critical roles after central nervous system injuries such as stroke or multiple sclerosis [5], [28]. To our knowledge, this is the first report that studies the frequency and functional status of circulating DCs in SAH patients. An immune dysfunction characterised by a profound and prolonged decreased number of circulating DCs (mDCs and pDCs) coupled with a decreased capacity of DCs in producing cytokines after specific TLR stimulations was observed.

Many features of critically-ill associated immunosuppression have already been described including: a decreased maturation of antigen presenting cells [29] and alteration in cytokine production by peripheral blood mononuclear cells [4], [20]. Apart from IL-6 and to a lesser extent IL-8, cytokine blood levels are surprisingly normal in SAH patients compared with healthy controls, indicating that SAH may exert some immunosuppressive effects. This apparent weak *in vivo* production of cytokines in SAH patients is still incompletely understood and could be explained either by alteration in cell reactivity to danger-associated molecular patterns through alteration in nuclear translocation of transcription factor such as NF-κB [30] or by a decrease in the number of circulating cytokine producing cells.

Alterations of DCs, one of the main sources of cytokines among peripheral blood mononuclear cells, have already been described in ICU patients. Grimaldi *et al.* reported a prolonged decrease of circulating myeloid and plasmacytoid DCs in septic patients [15]. Interestingly, the importance of this alteration was correlated with the occurrence of a secondary infection. A drastic decrease in the number of splenic DCs has recently been reported in patients succumbing from septic shock in an intensive care unit [31]. In accordance with these data obtained in critically ill patients, we currently report a major decrease of both myeloid and plasmacytoid DCs lasting up to 10 days in SAH patients. The observed reduction in circulating DCs could be related to a reduction in DC differentiation from bone marrow progenitors, with a diminished half-life of DCs or with the recruitment of circulating DCs to lymphoid organs or peripheral tissues [16], [32]. Since DCs are specialised in early pathogen detection, the decrease of circulating DCs could be a major feature of immunosuppression, explaining at least in part, the high incidence of pneumonia after SAH.

As for mDCs, SAH-induced alteration in cytokine production after TLR-3 and -4 stimulation predominantly affects IL-12. This cytokine is critical for inducing T-helper-cell response and defence of the host against intracellular pathogens [33]. Since mDCs are a major source of IL-12 in bacterial challenge [34], our results reveal a new determinant of SAH-induced immunosuppression. TLR-3 impairment is particularly spectacular in mDCs. Several hypotheses can be proposed to explain these functional alterations in TLR signalling in SAH patients. First, SAH could induce a rapid increase of sterile α- and armadillo-motif-containing protein (SARM) expression, which selectively alters TLR-3 and TLR-4 signalling pathways [35]. Indeed, SARM was shown to block the activation of TLR-3 and -4/TRIF-dependent transcription-factor and gene induction, without affecting TLR/MyD88-dependent pathway [36]. Second, SAH could induce an aberrant stimulation of the CD1c (BDCA-1)+ mDCs subset and a down-regulation of the CD141 (BDCA-3)+ subset. In fact, among mDCs, CD141 (BDCA-3)+ DCs express a restricted pattern of TLR with strong expression of and responsiveness to TLR-3 which was shown to induce IL-12 production [37]. CD1c+ DCs were shown to produce small amounts of IL-12 and TNF-α but display an immuno-regulatory phenotype through the production of IL-10 and of the regulatory molecules IDO and soluble CD25 [38]. IL-10 is not elevated in the current results, suggesting that the molecules IDO or soluble CD-25 could play a role in the observed mDCs alterations. Since the description of the CD1c subset is recent, we did not differentiate these two subsets.

Regarding pDCs, SAH patients have a long-lasting decreased capacity to produce IFN-α as well as TNF-α on TLR-7 stimulation. This inhibition is specific because the TLR-9 pathway remains functional. TLR-7/8 and 9 both activate a MyD88 pathway leading to the nuclear translocation of the NF-kB (TNF-α production), and the IRF (IFN-α production) families of transcription factors [39]. As TLR-7/8 and 9 share the same intracellular pathways, our results were quite unexpected. In the setting of neurological disease, the described abnormalities also appear to be specific, since with multiple sclerosis, the TLR-9 pathway was impaired [28]. Two hypotheses could be proposed to explain this pDC hyporeactivity. First, the transcriptions of TLR 7 and TLR 8 were shown to be reduced in peripheral blood mononuclear cells from stroke patients compared with healthy controls [40]. Second, acute brain injury may induce the rapid release of IL-10 [41]. However, the circulating IL-10 level is low, indicating that IL-10 is probably not involved in the immunodepression described in the present results. Finally, the inability of pDCs to produce IFN-α after TLR stimulation may prove important since pDCs cross-regulate mDC maturation through IFN-α, and alteration in maturation of immune cells has been demonstrated to be a key factor for outcome after brain injuries [4]. Moreover, in exploratory analysis, non-survivor patients have a decreased ability to produce IFN-α after TLR-9 stimulation compared with survivors.

Brain injuries induce the release of anti-inflammatory mediators, explaining the long-lasting immunosuppression generally observed after stroke or SAH [6]. The most common hypothesis is that this direct immunosuppresssive response of the brain occurs independently of the systemic pro/anti-inflammatory response. Surprisingly, there is very little evidence in the current literature to validate this attractive hypothesis. In this setting, the present results show that severe alterations of DCs occur on day 2 when the systemic inflammatory response appears to be weak (low CRP and blood cytokine levels). To the best of our knowledge, these data suggest for the first time that brain injury-induced immunosuppression is related to brain damage rather than to feedback from peripheral circulation.

Our study has some limitations. This preliminary study included a small cohort of patients and no statistical power analysis could have been performed. No definitive conclusion can thus be drawn from these results regarding the predictive value of TLR hypo-reactivity in DCs for death. Also, the alterations observed in DCs are probably not specific to BI but also observed in others populations of ICU patients. The analyses presented here could be somehow considered as essentially descriptive. However, circulating DCs represent a very low number of cells in the overall peripheral blood mononuclear cells, rendering any functional analysis complex. Finally, circulating DCs represent a very low number of cells in the overall peripheral blood mononuclear cells, rendering any functional analysis complex. This may explain that very few studies have investigated cytokine production in circulating DCs after TLR stimulation.

In summary, we describe a prolonged decrease in the number of circulatory mDCs and pDCs in a cohort of SAH patients. Our data also show selective alterations in the TLR reactivity of circulating DCs which could participate in SAH-induced immunosuppression. Since TLR agonists have recently been proposed for the prevention of infection in humans [42], the functional evaluation of TLR-signalling in DCs opens a novel cognitive research avenue for patients at high risk of nosocomial pneumonia who could benefit from therapies designed to restore the function of DCs [43].
